# Electrocardiogram-based detection of an atrial cardiomyopathy predicts postoperative supraventricular arrhythmias after lung transplantation

**DOI:** 10.1016/j.xjon.2025.09.028

**Published:** 2025-09-27

**Authors:** Paula C. Kuss, Björn C. Frye, Ina Hettich, Sebastian Fähndrich, Daiana Stolz, Martin Eichenlaub

**Affiliations:** a Clinic of Pneumology, Medical Center, University of Freiburg, Freiburg, Germany; b Department of Cardiology and Angiology, Medical Center, University of Freiburg, Bad Krozingen, Germany

**Figure:**
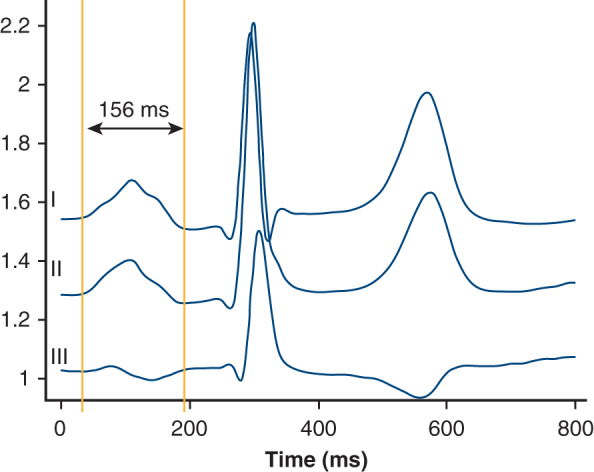
Measurement of amplified P-wave duration as surrogate for atrial cardiomyopathy.

**Central Message**

Prolonged amplified P-wave duration as marker of atrial cardiomyopathy predicts postoperative supraventricular arrhythmias after lung transplantation, leading to longer ICU and hospital stays.

Postoperative supraventricular arrhythmias (SVAs) occur frequently after lung transplantation (LTX) and significantly impact morbidity and mortality.^1^ Previous studies hypothesized that the pathogenesis of SVAs after LTX might be a multifactorial process involving an underlying atrial cardiomyopathy (AtCM) and various trigger factors (eg, inflammation, surgical stress).^2^ The measurement of amplified P-wave duration (aPWD) on digital electrocardiography (ECG) allows noninvasive quantification of AtCM and thus may aid the identification of this major risk factor for SVAs in vulnerable cohorts like patients undergoing LTX.^3^^,^^4^

## Methods

We retrospectively analyzed patients who underwent their first LTX at the University Hospital of Freiburg between 2014 and 2022 for available digital ECGs in sinus rhythm prior to LTX and a postoperative survival of at least 1 month. aPWD was measured after ECG amplification from standard settings (10 mm/mV and 25 mm/s) to 80 mm/mV and 175 mm/s by 2 independent investigators blinded to patients' outcome and characteristics as described previously by our group.^3^^,^^4^ The average of both measurements was used to calculate aPWD. We also assessed the COM-AF (combined atrial fibrillation [AF] risk score) score, a validated risk score for predicting postoperative SVAs after thoracic surgery consisting of age, female sex, diabetes, arterial hypertension, heart failure, and stroke.^5^ SVAs were defined according to current guidelines as any episode of AF, atrial flutter, or left atrial tachycardia lasting ≥30 seconds, recorded during telemetry monitoring on an intensive care unit (ICU) or a 12-lead ECG on a general ward. The study was approved by the hospital's Institutional Ethics Committee (24-1568-S1-retro; approved March 4, 2025). Owing to the study's retrospective design, informed consent was waived.

### Statistical Analysis

Statistical analysis was conducted using SPSS version 23 (IBM). The Shapiro-Wilk test was applied to assessed normality. Normally distributed data are presented as mean ± standard deviation; non-normally distributed data, as median with interquartile range. The Student *t* test, Mann-Whitney *U* test, and Fisher exact test were used to compare different groups. Receiver operating characteristic curve analysis determined the optimal aPWD cut-off for predicting SVAs. All independent variables were first evaluated using univariate linear regression analysis to identify potential predictors of postoperative SVAs. Variables of clinical relevance, including sex, number of cardiovascular risk factors, Lung Allocation Score (LAS), surgical procedure type and duration, and aPWD, were subsequently incorporated into a multivariable logistic regression analysis. A 2-tailed *P* value < .05 was considered statistically significant.

## Results

**Figure 1:**
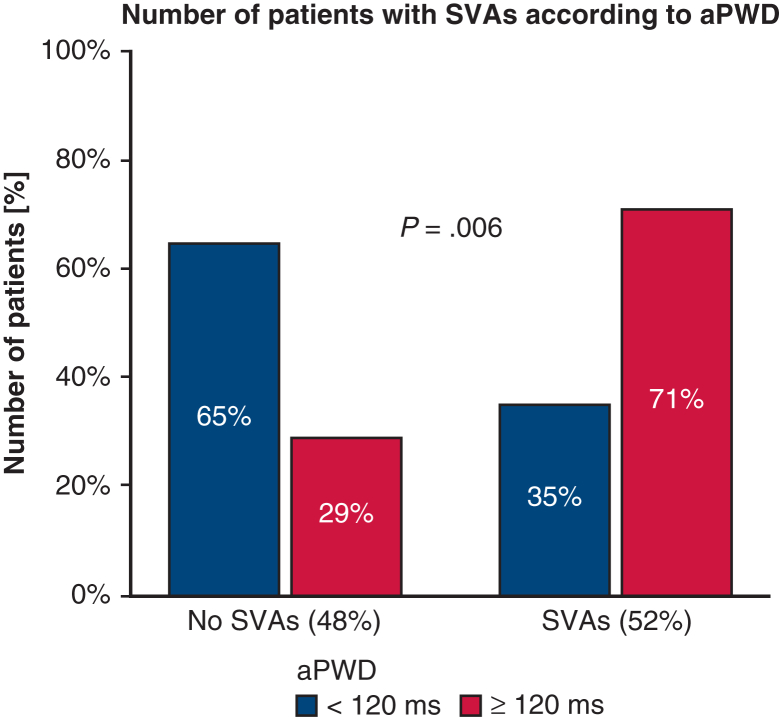
Number of correctly classified patients according to amplified P-wave duration (*aPWD*) between patients without and with supraventricular arrhythmias (*SVAs*).

**Figure E1:**
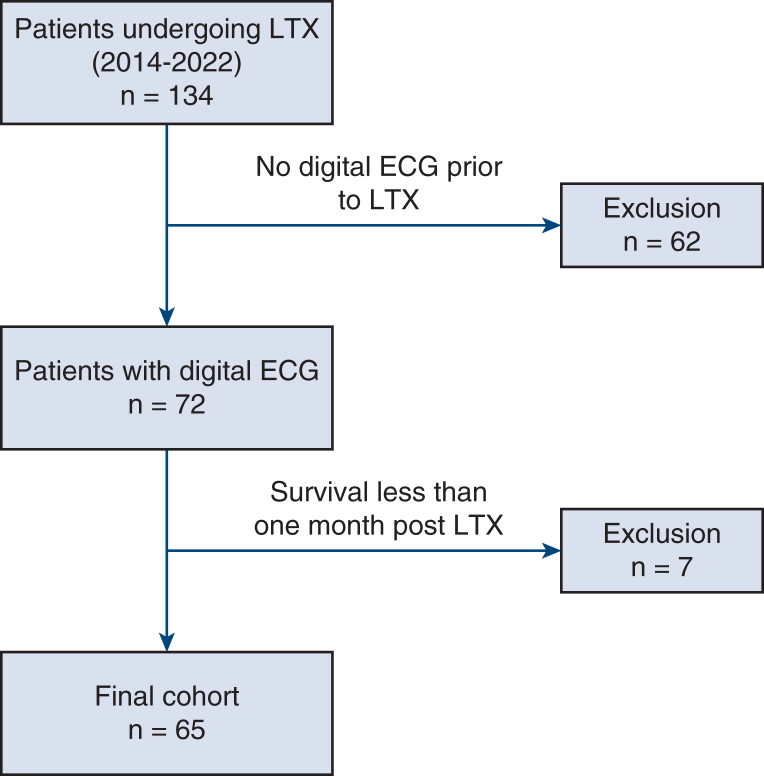
Overview of the overall cohort and the study population. *LTX*, Lung transplantation; *ECG*, electrocardiography.

**Figure E2:**
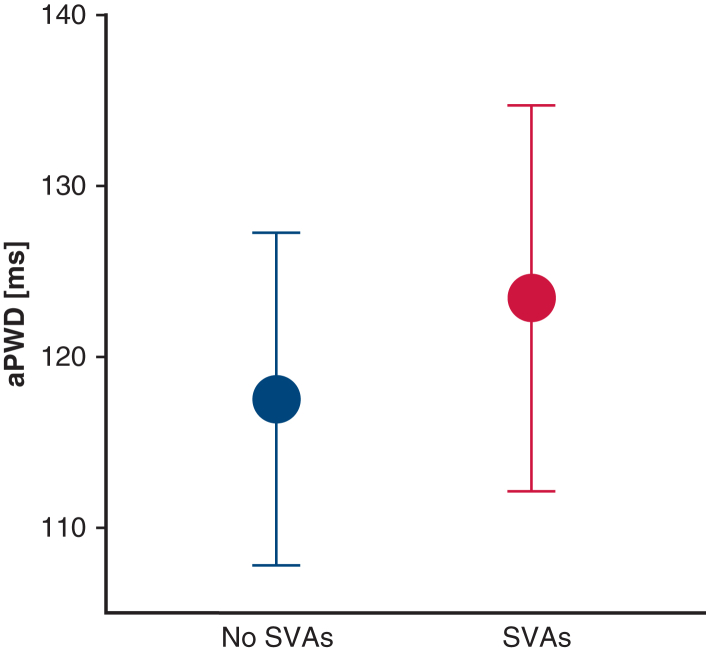
Comparison of amplified P-wave duration (*aPWD*) between patients with and without postoperative supraventricular arrhythmias (*SVA*). Bar plots show the groupwise mean ± standard deviation.

At total of 134 patients were identified as first LTX recipients between 2014 and 2022. Of these, 65 patients were included in our analysis (Figure E1). Clinical characteristics are displayed in Table 1. The median age was 60 years (interquartile range, 54.0-63.5 years), and 52.3% were male. The majority (95.4%) underwent double LTX, with chronic obstructive pulmonary disease the main indication (47.7%). Postoperative SVAs occurred in 34 patients (52.3%). Patients with SVAs had a significantly prolonged aPWD compared to those without SVAs (mean, 123 ± 11 ms vs 118 ± 10 ms; *P* = .03; Figure E2) prior to LTX. An aPWD of 120 ms was identified as the optimal cut-off, with a sensitivity of 65% and a specificity of 71% for SVA prediction and good discrimination between the 2 groups (Figure 1). COM-AF score ≥2 and left atrial diameter were not significantly different between patients with SVAs and patients without SVAs.

**Table 1:** Clinical and procedural characteristics

Characteristic	Total cohort (N = 65)	SVA (N = 34)	No SVA (N = 31)	*P* value
Baseline characteristics				
Age, y, median (IQR)	60.0 (54.0-63.5)	61.0 (56.5-63.3)	58.0 (53.0-64.0)	.14
Female sex, n (%)	31 (47.7)	16 (47.1)	15 (48.4)	1.0
BMI, kg/m^2^, mean ± SD	22.6 ± 4.2	22.8 ± 4.7	22.4 ± 3.8	.70
Comorbidities, n (%)				
Diabetes mellitus	11 (16.9)	3 (8.8)	8 (25.8)	.10
Hypertension	19 (29.2)	10 (29.4)	9 (29.0)	1.0
Hyperlipidemia	23 (35.4)	12 (35.3)	11 (35.5)	1.0
Coronary artery disease	12 (18.5)	7 (20.6)	5 (16.1)	.75
Pulmonary hypertension	19 (29.2)	10 (29.4)	9 (29.0)	1.0
COM-AF score ≥2 points	17 (26.2)	9 (26.5)	8 (25.8)	1.0
Smoking history				
Past smokers n (%)	45 (69.2)	25 (73.5)	20 (64.5)	.59
Pack-years, y, median (IQR)	25 (0-39)	30 (0-45)	10 (0-35)	.12
Number of CVRF, n (%)				
0	11 (16.9)	6 (17.6)	5 (16.1)	1.0
1	22 (33.8)	11 (32.4)	11 (35.5)	.80
2	22 (33.8)	12 (35.3)	10 (32.3)	1.0
3	8 (12.3)	5 (14.7)	3 (9.7)	.71
4	2 (3.1)	0	2 (6.5)	.22
SVA history, n (%)				
SVA prior to transplantation	7 (10.8)	4 (11.8)	3 (9.7)	1.0
Indication for transplantation, n (%)				
COPD	31 (47.7)	18 (52.9)	13 (41.9)	.46
Cystic fibrosis	4 (6.2)	1 (2.9)	3 (9.7)	.34
ILD	22 (33.8)	12 (35.3)	10 (32.3)	1.0
Other	8 (12.3)	3 (8.8)	5 (16.1)	.46
LAS, median (IQR)	36.7 (33.2-49.5)	35.1 (33.0-35.1)	38.2 (33.6-52.1)	.37
Type of LTX, n (%)				
Single lung	3 (4.6)	2 (5.9)	1 (3.2)	1.0
Double lung	62 (95.4)	32 (94.1)	30 (96.8)	1.0
Duration of surgery, min, mean ± SD	362 ± 90	376 ± 92	349 ± 88	.24
ECLS support, n (%)	18 (27.7)	10 (29.4)	8 (25.8)	.79
ECG findings, mean ± SD				
aPWD, ms	121 ± 11	123 ± 11	118 ± 10	**.03**
Echocardiographic findings				
LAD, mm, mean ± SD (N = 47)	33 ± 6	34 ± 6	32 ± 6	.24
LVEF, %	55 (55-60)	55 (55-59)	55 (55-60)	.28
LVEDD, mm	39.5 (5.1-47.0)	42.0 (29.0-49.0)	37.0 (4.7-44.0)	.136
Right heart catheterization				
Mean PAP, mm Hg, median (IQR)	23 (19-29)	25 (21-28)	23 (18-30)	.42
Mean PCWP, mm Hg, median (IQR)	9 (6-11)	9 (6-12)	8 (5-10)	.44
RAP, mm Hg, median (IQR)	5.0 (3.0-8.5)	6.0 (3.5-9.5)	4.0 (2.4-7.8)	.18
CO, L/min, mean ± SD	5.0 ± 1.0	5.0 ± 0.1	5.1 ± 1.1	.67
Cardiac index, L/min/m^2^, median (IQR)	2.8 (2.4-3.2)	2.8 (2.4-3.1)	2.9 (2.5-3.4)	.53
Treatment, n (%)				
Rate control	41 (62.1)	30 (88.2)	11 (35.5)	**<.001**
Beta blockers	40 (61.5)	29 (85.3)	11 (35.5)	**<.001**
Digitalis	3 (4.6)	3 (8.8)	0	.24
Verapamil	1 (1.5)	1 (2.9)	0	1.0
Rhythm control	18 (27.7)	18 (52.9)	0	**<.001**
Amiodarone	6 (9.2)	6 (17.6)	0	**.025**
Flecainide	16 (24.6)	16 (47.1)	0	**<.001**
Need for vasopressor support	59 (90.8)	31 (91.2)	28 (90.3)	1.0
Electrocardioversion	8 (12.3)	8 (23.5)	0	**.005**
Anticoagulation	25 (38.5)	18 (52.9)	7 (22.6)	**.021**
Clinical outcome				
Tracheotomy, n (%)	17 (26.2)	11 (32.4)	6 (19.4)	.27
Time on ventilator, d, median (IQR)	2 (2-13)	2 (1-20)	2 (2-4)	.57
Hospital stay, d, median (IQR)	42 (35-52)	47 (37-56)	40 (31-43)	**.01**
ICU stay, d, median (IQR)	9 (6-22)	11 (8-29)	8 (5-12)	**.02**
30-d mortality, n (%)	20 (30.8)	13 (38.2)	7 (22.6)	.19

Bold type indicates statistical significance. *SVA*, Supraventricular arrhythmias; *IQR*, interquartile range; *BMI*, body mass index; *COM-AF* score, combined atrial fibrillation risk score; *CVRF*, cardiovascular risk factors including diabetes, hyperlipidemia, smoking, and hypertension; *COPD*, chronic obstructive pulmonary disease; *LAS*, lung allocation score; *ECLS*, extracorporeal life support; *ECG*, electrocardiogram; *aPWD*, amplified P-wave duration; *LAD*, left atrial diameter; *LVEF*, left ventricular ejection fraction; *LVEDD*, left ventricular end-diastolic diameter; *PAP*, pulmonary arterial pressure; *PCWP*, pulmonary capillary wedge pressure; *RAP*, right atrial pressure; *CO*, cardiac output; *ICU*, intensive care unit.

Regression analysis identified prolonged aPWD as a significant predictor for postoperative SVAs, with a 6% increase in SVA risk for every millisecond of aPWD (odds ratio [OR], 1.06; 95% confidence interval [CI], 1.00-1.11; *P* = .03) and a >4-fold increased risk in patients with aPWD ≥120 ms (OR, 4.5; 95% CI, 1.57-12.77; *P* = .005). aPWD (OR, 1.06; 95% CI, 1.00-1.12 per ms; *P* = .047) also remained the sole significant independent predictor for SVAs after adjustment for sex, patient comorbidity, and overall illness severity, as well as technical complexity of LTX (Table E1).

Patients with SVAs were significantly more frequently treated with rhythm and rate control (both *P* < .001) or underwent electrical cardioversion (*P* = .005). Furthermore, they had significantly longer ICU (11 days vs 8 days; *P* = .02) and overall hospital (47 days vs 40 days; *P* = .01) lengths of stay. Mortality was numerically increased in patients with SVAs (38.2% vs 22.6%; *P* = .19).

## Discussion

In the present study, the incidence of postoperative SVAs following LTX was 52.3%, a higher rate than reported previously.^1^ This increased incidence likely can be attributed to the relatively high LAS in our patient population; the frequent performance of double LTX, leading to increased surgical stress; and the routine use of vasopressors post-LTX in our cohort, each of which is a significant trigger for SVAs.^E1^ Additionally, our strict definition of SVAs, including both episodes lasting ≥30 seconds on telemetry monitoring or 12-lead ECG, contrasts with other studies that considered only episodes recorded on 12-lead ECG.

AF typically originates from the pulmonary veins (PVs). Analogous to catheter-based pulmonary vein isolation (PVI), especially double LTX leads technically to a kind of surgical antral PVI.^E2^ However, paradoxically, the incidence of SVAs (especially AF) post-LTX is increased, emphasizing that an atrial substrate rather than PVs might be the origin of SVAs after LTX, especially in older patients and patients with left atrial enlargement. This substrate reflects a vulnerable myocardium, triggered by various stressors such as surgical stress, hemodynamic changes, and pain-induced overstimulation.^2^ Regardless of LTX, AtCM is an established risk factor for new-onset SVAs. Previous studies from our group demonstrated that an interatrial conduction delay caused by AtCM in AF patients can be quantified noninvasively by aPWD analysis.^3^^,^^4^ In this study, prolonged aPWD emerged as significant risk factor for postoperative SVAs in LTX recipients, maintaining its significance even after adjustment for sex, patient comorbidities and illness, and the technical complexity of the transplantation procedure, with a 6% increase in SVA risk for every millisecond of aPWD. An aPWD cut-off ≥120 ms was identified to predict SVAs with the best sensitivity and specificity and was associated with a more than 4-fold increased risk of SVAs.

In contrast to previous studies including symptomatic AF patients undergoing PVI, where aPWD cut-offs ranged from 140 to 150 ms, the threshold identified in our LTX cohort was notably shorter, likely reflecting the lower extent of AtCM in a cohort predominantly consisting of patients without history of prior AF.^4^ Interestingly, left atrial diameter as another surrogate marker of AtCM did not differ significantly between patients with and without SVAs, and the correlation between aPWD and left atrial diameter was only weak (r = 0.03; *P* = .82). This may be explained on the one hand by the fact that left atrial diameter measurements can yield imprecise data if the orientation of the long axis of the left atrium is not optimal and true orthogonal diameters cannot be obtained, and on the other hand by the fact that this parameter reflects only left atrial dilatation and not left atrial function. Nevertheless, the hypothesis that AtCM plays a major role in the development of postoperative SVAs is further supported by the observation that among the 7 patients with preoperative AF, those who experienced postoperative SVAs had a numerically longer aPWD (mean, 120 ± 14 ms; n = 4) compared with those who did not (mean, 110 ± 6 ms; n = 3), although the difference was not statistically significant (*P* = .24). If the source of preoperative AF in these patients was attributable to the PV substrate, the “surgical PVI” performed as part of the LTX procedure appeared to be protective, whereas AF due to AtCM (as indicated by a longer preoperative aPWD) was more likely to persist. These assumptions should be interpreted with caution, however, given the small number of patients in this subgroup.

Consistent with previous studies, we demonstrated that patients with SVAs had significantly longer ICU and the overall hospital lengths of stay, which is associated with higher costs.^1^ Thus, preoperative identification of patients at increased risk for SVAs might enable closer perioperative monitoring and possibly prevention by using antiarrhythmic drugs.

### Limitations

The main limitations of this single-center proof-of-concept study are its retrospective design, small sample size of 65 patients, and lack of a validation cohort. Inclusion was restricted to patients with a digital ECG in sinus rhythm before LTX, potentially causing selection bias. Nevertheless, our robust findings despite the small sample size and modest difference in mean aPWD support the concept of aPWD as a surrogate marker of AtCM predisposing to SVA.

## Conclusions

Detection of AtCM diagnosed by prolonged aPWD allows the identification of patients at increased risk for SVAs after LTX who merit closer monitoring. The clinical impact is demonstrated by the significantly longer both ICU and overall hospital lengths of stay in patients with SVAs.

## Conflict of Interest Statement

Dr Frye reported lecture and advisory fees from Advita Lifescience, Astra Zeneca, Boehringer Ingelheim, and Vifor; research grants from Advita Lifescience and BMS; and support from the Berta-Ottenstein-Program for Advanced Clinician Scientists from the Faculty of Medicine, University of Freiburg. Dr Fähndrich reported fees for lectures and travel support from CSL Behring, Grifols, AstraZeneca, and BerlinChemie and research support from the German Federal Ministry of Education and Research (BMBF) and the European Union (ISIDORe). Dr Stolz reported payments or honoraria for lectures, presentations, advisory boards, speakers' bureaus, manuscript writing or educational events from AstraZeneca, Berlin-Chemie/Menarini, Boehringer Ingelheim, Chiesi, CSL Behring, Curetis, GSK, Merck, MSD, Novartis, Roche, Sanofi, and Vifor. All other authors reported no conflicts of interest.

The *Journal* policy requires editors and reviewers to disclose conflicts of interest and to decline handling or reviewing manuscripts for which they may have a conflict of interest. The editors and reviewers of this article have no conflicts of interest.

## Figures and Tables

**Table E1:** Multivariable regression analysis for SVA prediction

Variable	OR (95% CI)	*P* value
aPWD	1.06 (1.00-1.12)	**.047**
LAS	0.98 (0.94-1.01)	.222
Duration of surgery	1.01 (1.00-1.02)	.059
Double LTX	0.28 (0.02-4.52)	.369
Male sex	1.02 (0.30-3.42)	.979
Number of CVRF	0.62 (0.32-1.21)	.161

Bold type indicates statistical significance. *SVA*, Supraventricular arrhythmias; *OR*, odds ratio; *CI*, confidence interval; *aPWD*, amplified P-wave duration; *LAS*, Lung Allocation Score; *LTX*, lung transplantation; *CVRF*, cardiovascular risk factors including diabetes, hyperlipidemia, smoking and hypertension.
